# Coverage and Effectiveness of Kyasanur Forest Disease (KFD) Vaccine in Karnataka, South India, 2005–10

**DOI:** 10.1371/journal.pntd.0002025

**Published:** 2013-01-24

**Authors:** Gudadappa S. Kasabi, Manoj V. Murhekar, Vijay K. Sandhya, Ramappa Raghunandan, Shivani K. Kiran, Gowdra H. Channabasappa, Sanjay M. Mehendale

**Affiliations:** 1 National Institute of Epidemiology (ICMR), Chennai, India; 2 Department of Health and Family Welfare, Government of Karnataka, Shimoga, India; 3 Virus Diagnostic Laboratory, Shimoga, Karnataka, India; Tulane School of Public Health and Tropical Medicine, United States of America

## Abstract

**Background:**

Kyasanur forest disease (KFD), a tick-borne viral disease with hemorrhagic manifestations, is localised in five districts of Karnataka state, India. Annual rounds of vaccination using formalin inactivated tissue-culture vaccine have been conducted in the region since 1990. Two doses of vaccine are administered to individuals aged 7–65 years at an interval of one month followed by periodic boosters after 6–9 months. In spite of high effectiveness of the vaccine reported in earlier studies, KFD cases among vaccinated individuals have been recently reported. We analysed KFD vaccination and case surveillance data from 2005 to 2010.

**Methodology/Principal Findings:**

We calculated KFD incidence among vaccinated and unvaccinated populations and computed the relative risk and vaccine effectiveness. During 2005–2010, a total of 343,256 individuals were eligible for KFD vaccination (details of vaccination for 2008 were not available). Of these, 52% did not receive any vaccine while 36% had received two doses and a booster. Of the 168 laboratory-confirmed KFD cases reported during this 5-year period, 134 (80%) were unvaccinated, nine each had received one and two doses respectively while 16 had received a booster during the pre-transmission season. The relative risks of disease following one, two and booster doses of vaccine were 1.06 (95% CI = 0.54–2.1), 0.38 (95% CI = 0.19–0.74) and 0.17 (95% CI = 0.10–0.29) respectively. The effectiveness of the vaccine was 62.4% (95% CI = 26.1–80.8) among those who received two doses and 82.9% (95% CI = 71.3–89.8) for those who received two doses followed by a booster dose as compared to the unvaccinated individuals.

**Conclusions:**

Coverage of KFD vaccine in the study area was low. Observed effectiveness of the KFD vaccine was lower as compared to the earlier reports, especially after a single dose administration. Systematic efforts are needed to increase the vaccine coverage and identify the reasons for lower effectiveness of the vaccine in the region.

## Author Summary

Kyasanur forest disease (KFD), a tick-borne viral disease with hemorrhagic manifestations, occurs as seasonal outbreaks in five districts of Karnataka state, India. Vaccination with formalin inactivated tissue-culture vaccine is the key strategy for the prevention of the disease in the region. In spite of high effectiveness of the vaccine reported in earlier studies, KFD cases among vaccinated individuals have been recently reported. We analysed KFD vaccination and case surveillance data from 2005 to 2010 to estimate the coverage and efficacy of the vaccine under programme conditions. Vaccination coverage was low with less than 50% of the population vaccinated. The effectiveness of the vaccine was 62.4% (95% CI = 26.1–80.8) among those who received two doses and 82.9% (95% CI = 71.3–89.8) for those who received an additional booster dose as compared to the unvaccinated individuals. Systematic efforts are needed to increase the vaccine coverage and identify the reasons for lower efficacy of the vaccine in the region.

## Introduction

Kyasanur forest disease (KFD) is a tick-borne viral disease characterised by sudden onset of fever and/or headache followed by hemorrhagic manifestations such as conjunctival congestion, bleeding gums, epistaxis, haemoptysis, haematemesis and malena [1]–[3]. The disease which was reported for the first time from Shimoga district of Karnataka state, India in 1957, is localised in five districts (Shimoga, Chikamagalur, Uttar Kannada, Dakshina Kannada and Udupi) of the state and occurs as seasonal outbreaks during December to May when the nymphal activity of ticks in the forest is maximum.

Prior to the currently used formalin inactivated KFD virus (KFDV) vaccine produced in chick embryo fibroblasts, several vaccines were tested for the control of the disease. These included 5–10% suspension of formalin-inactivated Russian Spring Summer Encephalitis (RSSE) virus [4]–[6], formalin inactivated vaccine from mice brain [7] as well as tissue culture source [8]–[9] and a live attenuated vaccine through serial tissue culture passages [10]. Field studies conducted in 1970–71 with the formolized KFDV demonstrated a serological response in 59% of the vaccinees after two doses [11]–[12].

Based on these findings, a vaccine production unit was established at Shimoga, Karnataka and the indigenously manufactured vaccine was licensed for use in the affected districts. Subsequently, the vaccine production was shifted to the Institute of Animal Husbandry and Veterinary Biologicals, Hebbal, Bangalore. Immunization with this vaccine has remained the key strategy for prevention of KFD in Karnataka since 1990. The focal immunization strategy involves annual rounds of vaccination using formalin inactivated tissue-culture vaccine. These campaigns are conducted during the months of August-November in the areas that reported KFD activity (defined as laboratory evidence of confirmed case/s in monkeys/humans or infected ticks) in the previous transmission seasons and surrounding villages within a radius of 5 Km [13]. Two doses of the vaccine are administered to individuals aged 7–65 years at an interval of one month. As the immunity conferred by the vaccination is short-lived, booster doses are recommended within 6–9 months after primary vaccination and repeated for five consecutive years after the last confirmed case in the area [13]. If cases of KFD are reported in the area in spite of vaccination during the pre-transmission season, additional vaccination campaigns are conducted. As part of the KFD vaccination programme, information about the number of individuals eligible for vaccination as well as coverage of vaccine is routinely compiled.

In the previous field evaluation of the vaccine during 1990–92 in Shimoga, Uttar Kannada and Chikamagalur districts, an effectiveness of 79.3% (95% CI: 64.7–87.9) with one dose and 93.5% (95% CI: 87.9–96.6) with two doses was reported [14]. However, despite routine vaccination, an increasing number of KFD cases have been reported during 1999–2005 suggesting sub-optimal efficacy of the current vaccine or vaccination protocol [15]. During the recent KFD outbreak in Shimoga in December 2011–March 2012, two doses of the vaccine given during April–May 2011 were found to confer no protection amongst the cases reported during Dec 2011–Mar 2012 [16]. With this background, we analysed the KFD vaccination and surveillance data from the five KFD endemic districts of Karnataka for the period 2005–2010 to estimate the coverage and the effectiveness of the KFD vaccine in the region.

## Methods

### KFD surveillance

As part of KFD surveillance in the region, health workers conduct door to door search for identifying suspected case-patients (defined as sudden onset of fever, headache and myalgia) in a radius of 5 km. surrounding the villages reporting recent monkey deaths or laboratory-confirmed KFD cases. Blood samples are collected from all the suspected case-patients and are tested for the nested polymerase chain reaction (RT-PCR) [17] and/or intra-cerebral inoculation of the sera into suckling mice. Information about number of doses of the KFD vaccine received is also collected from the case-patients. We reviewed the KFD vaccination and the surveillance data for the year 2005–2010 from the five KFD endemic districts.

### Statistical methods

We calculated the annual and overall incidence of KFD during 2005–10 among the vaccinated population and compared the same with the incidence of the disease among unvaccinated individuals to calculate the relative risk (RR) associated with vaccination. Using Epi-6 software (CDC, Atlanta), we calculated the effectiveness of the vaccine (VE) and its 95% confidence intervals (CI).

### Human subject protection

The study primarily involved analysis of the archived data of the KFD vaccination programme and surveillance conducted by Karnataka state health department. Permission from the district health authorities was obtained to access the data.

## Results

During 2005–10, a total of 343,256 individuals from the five districts were eligible for KFD vaccination as these individuals resided in villages within 5 km radius of villages that reported monkey deaths or a laboratory confirmed human case of KFD or tick positivity. The vaccination details for 2008 were not available and hence were not included in the calculation of vaccine effectiveness.

About 52% of the eligible population did not receive any vaccine while 36% of the population received two doses and a booster dose (Table 1). The remaining 12% of the population received one or two doses of the vaccine. A total of 168 laboratory confirmed KFD cases were reported during this five year period of which 134 (80%) were unvaccinated. Of the remaining 34 cases, nine each had received one and two doses respectively while 16 had received a booster in the pre-transmission season. The incidence of KFD among the individuals who had received one dose of the vaccine was similar to the incidence among those who were unvaccinated (8 per 10,000 in both groups; RR: 1.06, 95% CI: 0.54–2.1). Compared to unvaccinated individuals, the incidence was significantly lower among the individuals who received two doses (3 per 10,000, RR: 0.38, 95% CI: 0.19–0.74) or two doses and booster (1 per 10,000, RR: 0.17, 95% CI: 0.10–0.29) dose of the vaccine. The effectiveness of the vaccine was 62.4% (95% CI: 26.1–80.8) among those who received two doses and 82.9% (95% CI: 71.3–89.8) for those who received two doses and a booster dose as compared to unvaccinated individuals.

**Table 1:** Incidence of KFD by number of doses of vaccine, Karnataka, 2005–10.

Year	Period of vaccination campaign	Target population	Number of doses of KFD vaccine received				Number of KFD cases (incidence per 10,000)			
			0 doses	1 doses	2 doses	2 doses+1 booster	0 doses	1 doses	2 doses	2 doses+1 booster
2005	Aug 2005–Apr 2006	136,102	75,996	8,053	14,468	37,585	71 (9)	9 (11)	3 (2)	16 (4)
2006	Aug 2006–Sept 2007	71,397	59,902	1,055	391	10,049	8 (1)	0 (0)	6 (153)	0 (0)
2007	Dec 2007–Mar 2008	83,432	33,529	1,245	9,661	38,997	36 (11)	0 (0)	0 (0)	0 (0)
2009	Aug 2009–Feb 2010	28,105	5,491	213	2,574	19,827	0 (0)	0 (0)	0 (0)	0 (0)
2010	Oct 2010–Jul 2011	24,220	2,095	623	4,493	17,009	19 (91)	0 (0)	0 (0)	0 (0)
Total		343,256	177,013 (52%)	11,189 (3%)	31,587 (9%)	123,467 (36%)	134 (8)	9 (8)	9 (3)	16 (1)

(Complete data for 2008 was not available and hence not included in the analysis).

## Discussion

The findings of our analysis of surveillance and vaccination data indicated lower effectiveness of the vaccine as compared to the earlier report of effectiveness of 79% and 94% with one and two doses of the vaccine respectively [14]. In particular, the administration of one dose was found to be non-efficacious as the incidence of the disease among such individuals was comparable with unvaccinated individuals. Our findings of lower effectiveness of the vaccine corroborate well with earlier reports of lower efficacy by other investigators [15]. It is pertinent to note that the breakthrough cases of KFD occurred only during 2005 and 2006 and there were no cases among the vaccinated individuals in subsequent years. The breakthrough cases were reported from three of the five districts.

Several reasons have been postulated for the lower efficacy of the vaccine including the possibility of drifts and diversity in the recently currently circulating strains of the KFD virus in contrast to the strain used for vaccine preparation (isolated in 1950s) [15]. However, the genotyping studies conducted on 48 KFD viruses isolated over the past five decades showed a low level of diversity with a maximum of 1.2% nt and 0.5% aa differences seen among these viruses [17]. It is necessary to evaluate other reasons for lower efficacy of the vaccine including the issues related with cold chain maintenance.

The coverage of the vaccine in the region was low with nearly half of the target population being unvaccinated. About 45% of the population received two doses or two doses and a booster, which were found to be protective. Achieving high coverage with two doses and/or booster doses of vaccine appears to be the key for the control of KFD in the endemic districts.

Our study had certain limitations. First, we used the available surveillance and programmatic data to estimate the coverage and the effectiveness of the vaccine. The number of KFD cases reported in the surveillance system is likely to be affected by its notification efficiency. However as the awareness about the disease in the area is high and most of the cases seek treatment from public health facilities in the area, we believe that the surveillance data reflects the true situation of KFD in the area and the number of missed cases is likely to be negligible. Second, the vaccination coverage estimated using administrative method in our analysis is likely to be higher than the actual coverage estimated through surveys.

In conclusion, the coverage of KFD vaccine in the five KFD endemic districts of Karnataka was low. The effectiveness of the vaccine was also found to be lower as compared to the earlier studies especially among those who received single dose of the vaccine. Systematic efforts are needed to increase the coverage of the vaccine among the areas targeted for vaccination to more effectively control this well-localized spread of KFD virus in Karnataka. It is also necessary to understand the reasons of lower uptake of the vaccine. The vaccine associated side effects such as pain as well as the number of doses to be taken over a period of five years are some potential deterring factors. Epidemiological studies are also needed to assess the long-term protection offered by boosters. Future research needs to focus on further refinement of the vaccine candidate to eliminate these deficiencies to make the vaccine safer and more effective in order to avoid the need for periodic boosters.
